# Supplementary material for: Manufacturing of All Inkjet-Printed Organic Photovoltaic Cell Arrays and Evaluating Their Suitability for Flexible Electronics
Source: Micromachines (Basel). 2018 Dec 4;9(12):642. doi: 10.3390/mi9120642 (PMC6316076; doi:10.3390/mi9120642)
Supplement: Supplementary file 1 [file micromachines-09-00642-s001.pdf]

Supplementary Materials: Manufacturing of All Inkjet-Printed Organic Photovoltaic Cell Arrays and Evaluating their Suitability for Flexible Electronics

Kalyan Yoti Mitra, Abdelrahman Alalawe, Stefanie Voigt, Christine Boeffel and Reinhard R. Baumann

Figure S1 shows first a photograph of an inkjet-printed P3HT:PCBM layer deposited using the setting described in Table 1 of the main manuscript. The printed rectangular patterns were thoroughly investigated using optical microscopy at two locations, the center (Figure S1B) and the edge (Figure S1C). From both the microscopic images, it can be seen clearly that due to the implication of strong pinning and a coffee ring effect, there were always occurrences of steps or wavy contours at regular intervals, generated from an inward to an outward direction. Finally, there was a dense ring-shaped accumulation at the center of the printed layer. To accomplish functional inkjet-printed OPV devices, it is very crucial to know the continuity of the printed BHJ layer and its reproducibility with such wavy characteristics. Therefore, laser scanning microscopy was performed over a measurement area of 25 µm × 1268 µm at the center location of the printed layers (marked with the orange color), where the steps or wavy-shaped contours were found to be mostly prominent. The results showed that the waves generated from the inconsistent drying phenomenon led to recurring steps, positioned at every 150 µm. The value of the highest peak frequently reached 300 nm from the surface of the valley, which was about 70 nm thick from the substrates. This indicates that the printed layers were fairly inhomogeneous, but they were continuous and pinhole-free, with a minimum layer thickness of about 70 nm.

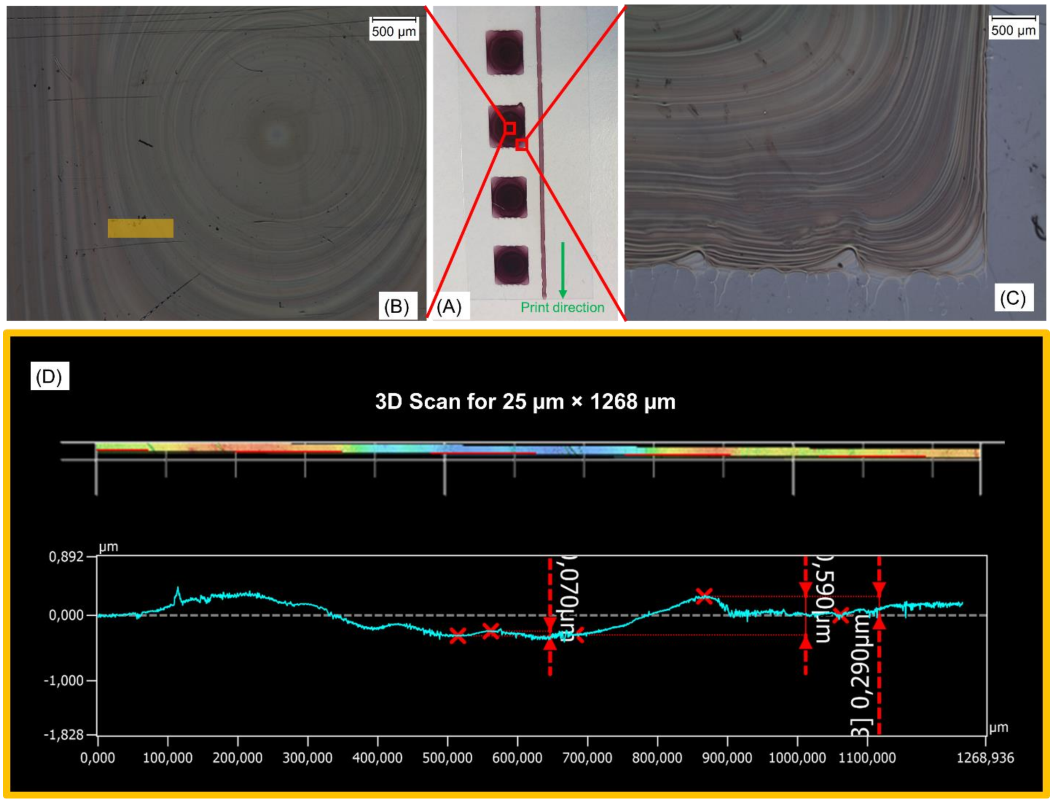

Figure S1. **(A)** Photographic and **(B–C)** microscopic images (optical devices) of the inkjet-printed P3HT:PCBM layer on PEN substrate, and **(D)** an image showing the result from the characterization performed using a laser microscopy from a specific "orange" location that is marked in (**B**).

Similar results can also be seen in Figure S2, where the topological characteristics are exhibited for the ZnO-based ETL layer, on the printed Ag electrode. Here, the layer was also found to be fairly inhomogeneous at the outer edges, but it was continuous and pinhole-free at the center (location of active area) with a minimum layer thickness of about 70 nm above the Ag layer and 300 nm as the nominal height of the generated steps or wave contours. The printed Ag layer, on the other hand, was found to be homogenous, with a thickness of about 300 nm.

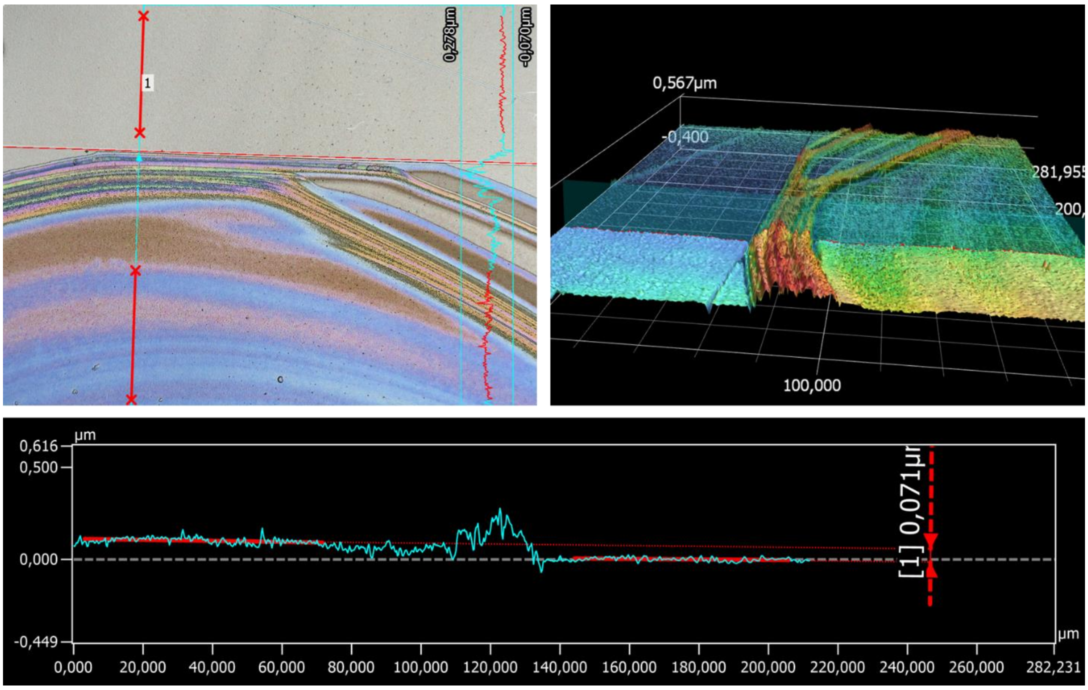

Figure S2. Results of the microscopic characterization performed over the edge of a printed ZnO-based ETL layer deposited on the previously deposited Ag layer.

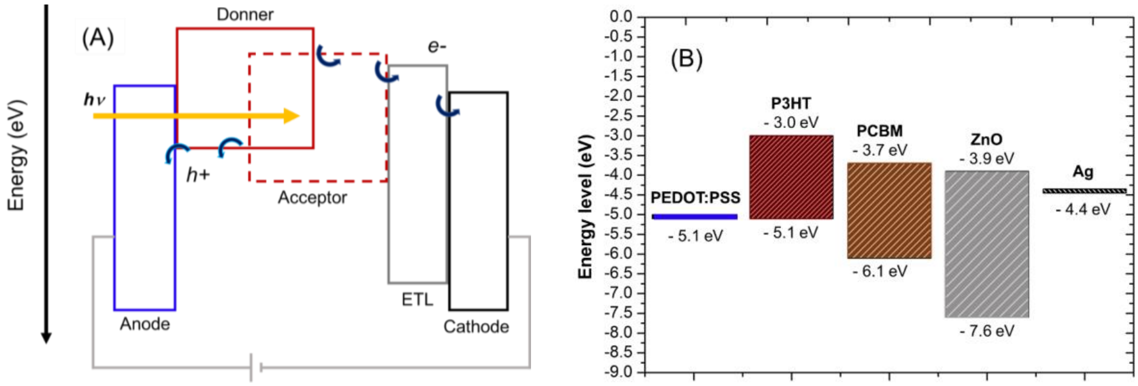

Figure S3. Graphs corresponding to **(A)** the charge transport mechanism of the electron hole pair for the working of the OPV stack and **(B)** the energy level diagram for the implemented functional material layers.
